# Endemic plants of Crete in electronic trade and wildlife tourism: current patterns and implications for conservation

**DOI:** 10.1186/s40709-019-0104-z

**Published:** 2019-10-30

**Authors:** Viktoria Menteli, Nikos Krigas, Manolis Avramakis, Nicholas Turland, Despoina Vokou

**Affiliations:** 1 Department of Ecology, School of Biology, Aristotle University of Thessaloniki, 54124 Thessaloniki, Greece; 2 Institute of Plant Breeding and Genetic Resources, Hellenic Agricultural Organization Demeter, P.O. Box 60458, 57001 Thessaloniki, Greece; 3 Natural History Museum of Crete, University of Crete, 71409 Heraklion, Greece; 4 Botanischer Garten und Botanisches Museum Berlin, Freie Universität Berlin, Königin-Luise-Str. 6-8, 14195 Berlin, Germany

**Keywords:** Botanical tours, Conservation, Crete, Cretan endemics, Cyprus, E-trade, Plant monitoring, Endemic plants

## Abstract

**Background:**

The island of Crete is a biodiversity hotspot having 223 endemic vascular taxa (species and subspecies) as a result of its long isolation and the wide range of habitats it includes. We explore trends and patterns in the electronic trade of these unique genetic resources and in their involvement in wildlife tourism, the ways these two activities are performed and the associated potential threats on the plants’ wild populations, and we also identify priority taxa requiring special attention. The main part of the study was conducted in 2016–2017 using English as a search language; an additional search was conducted in 2019 using German and French.

**Results:**

We found e-commerce for 28 (13%) endemic taxa. These are traded by 65 nurseries from 14 countries, the UK primarily. Among the traded plants, 16 face extinction risk and/or are under protection status. Prices vary largely for the same taxon and form of sale. Lamiaceae is the family with the highest number of e-traded taxa, *Tulipa bakeri* is the most traded species, and the living plant is the commonest form of sale. Thirty-seven endemic taxa are advertised in the websites of travel agencies involved in wildlife tourism. *Tulipa doerfleri* is the most frequently encountered taxon in these websites, whereas Lamiaceae, Liliaceae and Orchidaceae are similarly represented. The additional search showed a very rapid increase in the e-trade of the Cretan endemis.

**Conclusion:**

The two examined markets are similar in that geophytes play a prominent role and Lamiaceae rank first among the represented plant families, but differ in several aspects: only 22.6% of the taxa detected are common in both, obedience to rules exhibited by travel agencies is not usually the case with nurseries, and potential threats to wild populations are estimated as considerably higher for the traded plants. Sixteen endemic taxa of Crete were identified as requiring special attention.

## Background

Crete, an Eastern Mediterranean island, is the largest island of Greece and the fifth largest in the Mediterranean basin. Of an elongated shape, it has a diversified terrain, but it is predominantly mountainous, with three mountain massifs higher than 2000 m. Its topographic and climatic variability have created a wide range of habitats, which in combination with the island’s long isolation have resulted into high rates of plant endemism [1, 2]. The massifs of the island, in particular, host many endemic taxa [3], more than Mt Olympus, the highest mountain of Greece [4, 5]. Crete is recognized by IUCN as a Global Centre of Plant Diversity [6]. At the same time, it is facing high tourism pressure like most Mediterranean islands and intense anthropogenic activity [1].

Overexploitation of natural resources has been identified as one of the causes of biodiversity loss worldwide [7–9]. The rich and unique flora of Crete attracts scientists, tourists, amateur and professional botanists, collectors, traders (legal and illegal) and nursery holders from all over the world. Given this, the risk associated with the various activities involving the plant genetic resources of Crete should be evaluated before it is too late.

The importance of the internet in getting information and goods has grown dramatically over the last years and is expected to become remarkably bigger the years to come [10]. This is also the case for plants’ trade. Several organizations, such as the International Plant Protection Convention (IPPC), the World Trade Organization, the World Custom Organization and the Convention on Biological Diversity [11–14] have examined this form of trade from different aspects (plant protection, phytosanitary, biodiversity conservation). The importance of controlling this big market was underlined in the 12th Session of the Commission on Phytosanitary Measures (2017). Despite the importance of e-trade, there are only few assessments of e-traded plants. These concern plants of Greece [15] and Cyprus [16], or they focus on specific plant families like orchids [17]. Data on illegal trafficking of wild flora are also limited [18–26]. Nevertheless, Alacs and Georges [22] estimate that illegal trading of wildlife is worth of more than US $20 billion per year, not including the e-trade. At the same time, the Global Strategy for Plant Conservation 2011–2020 set the Target 11, emphasizing that no species of wild flora should be endangered by international trade.

In this study, which makes part of a wider project regarding the local endemic plants of major Mediterranean islands, we explore trends and patterns (i) in the electronic trade of these unique genetic resources of Crete (single-island endemics) and (ii) in their involvement in wildlife tourism; we also examine ways in which these activities are conducted that could affect their wild populations. More specifically, we answer to the following questions: which are the plant taxa that are traded worldwide as genetic resources (living material); which are the ones of high demand; are there plants facing extinction risk and/or are protected among them; do prices differ depending on the vulnerability/protection status of plants; which plants are most attractive to wildlife tourists; which are the preferred types of habitat in plant-oriented wildlife tourism; are the two activities, i.e. international e-trade of Cretan endemics and plant-oriented wildlife tourism in Crete, comparable in the practices that they follow, and what are the associated expected impacts on natural populations? Based on the above, we further aim at identifying priority plants that require special attention. For our study, we made use of the information provided via the internet in the English language. By doing so, we have a partial, yet representative, picture of the activities involving these plants.

## Methods

For our research, we updated the list of the local endemic flora of Crete [27] on the basis of the most informed literature on the flora of Crete and Greece [28–30]. We updated all scientific names of taxa using the currently accepted ones and we cross-checked endemism and chorology based on Dimopoulos et al. [28, 29]. After cross-checking, we excluded from the previous list: (a) all hybrids mentioned by Fielding and Turland [27]; (b) taxa that were not single-island endemics as they were found subsequently also on Karpathos island (*Arum purpureospathum*, *Bellis longifolia*, *Pimpinella tragium* subsp. *depressa*, *Hypericum empetrifolium* subsp. *oliganthum*) or elsewhere in Greece (*Carlina curetum*, *Galium graecum* subsp. *pseudocanum*, *Solenopsis minuta* subsp. *annua*) or in other countries (*Androcymbium rechingeri*) and (c) taxa that are now included under more widespread ones, i.e. *Centaurea raphanina* subsp. *saxatilis* nowadays under *C. raphanina* subsp. *raphanina*; *Cirsium creticum* subsp. *dictaeum* under *C. creticum* subsp. *creticum*; *Colchicum cousturieri* under *C. cupanii* subsp. c*upanii*; *Ophrys creberrima*, *O. cressa*, *O. creticola* and *O. thriptiensis*, all under *O. fusca* subsp. *fusca*; *Ophrys cretica* subsp. *bicornuta* under *O. cretica* subsp. *cretica*, and *O. grigoriana* under *O. sphegodes* subsp. *spruneri*. In our updated list of the local endemic plants of Crete, we added: (a) taxa that have been well-established and are therefore currently accepted [27], i.e. *Prospero battagliae*, *P. depressum*, *P. hierapytnense*, *P. idaeum* and *P. rhadamanthi*; (b) taxa that have been re-established after taxonomic revisions and are therefore accepted [28, 29], i.e. *Astragalus creticus* subsp. *creticus* and subsp. *minoicus*, *A. dolinicola* [31], *Carex cretica* [32, 33] and *C. idaea* [32], *Cotoneaster creticus* [34], *Cyclamen confusum* [35], *Festuca polita* subsp*. cretica* [36], *Hieracium schmidtii* subsp. *creticum* [37]*, Linum caespitosum* and *L. doerfleri* [38], *Micromeria hispida* and *M. sphaciotica* [39], and *Tulipa bakeri* [27–30, 40]; and (c) taxa that have been recently discovered and described [41–50]. This extensive update after the most recently published information resulted in 223 taxa that make nowadays the endemic flora of vascular plants of Crete (species and subspecies) (see Additional file 1).

For every single-island endemic taxon, we searched for indications of electronic trade as living material (whole plant, bulb or seed). We did not consider trade of dried herbal material cut from plants (wild growing or cultivated) that is used for tea making (i) because in this form, plants are not genetic resources, and (ii) because an exploratory study that we conducted before finalizing the methodology of our research showed that the single-island endemic plants of Crete contained in these teas are almost exclusively *Origanum dictamnus*, *Sideritis syriaca* subsp. *syriaca* and *Origanum microphyllum*. All three derive from small-scale cultivations or from the wild, in the latter case being collected under special permits issued by the Greek authorities following assessment of the natural populations, and are certified.

We run this search through two main websites that are linked with a large number of nurseries. These are the Plant Finder application (https://www.rhs.org.uk/plants/search-form) of the Royal Horticultural Society webpage, which provides information for plants traded mostly in European nurseries, and the Plant Information Online webpage (https://plantinfo.umn.edu/), which provides data for nurseries in the USA and Canada [16]. All information was checked thoroughly, as described in Krigas et al. [15]. We confirmed the trading indication in these platforms by checking the updated webpage of every nursery mentioned. Furthermore, we did a Google search during the same period. For every endemic taxon, we searched using two phrases: “buy + scientific name” and “sell + scientific name”. In case there were synonyms of the plant taxa examined, we repeated the search following the same procedure. Also, a combined search with all names (accepted and synonyms) was conducted. We recorded the information from all relevant sources included in the first 10 pages (100 results) of the search output [16]. This is the limit we set, after several preliminary searches, which showed that the first 10 pages contain more than 90% of the sources of relevance appearing in the 50 pages searched. We conducted all the above searches during the period July 2016–July 2017.

We listed the nurseries’ websites, the forms of trade (seeds, bulbs and living plants), selling prices and currency and the countries where nurseries are located. We converted all currencies to Euros (€) and US Dollars (USD) according to the monetary rates on July 17, 2017 (1 € = 1.15 USD). We recorded the extinction risk status of each endemic taxon according to the latest assessment [51–55]. Also, we examined whether the traded taxa are included in the Annexes II and IV of the Habitats Directive (92/43/EEC) or in the Bern Convention or in the Convention on International Trade in Endangered Species of Wild Fauna and Flora (CITES) and also, at the national level, in the Presidential Decree 67/81 (Governmental Gazette 23/Α/81) for the protection of native flora and wild fauna. To determine if the extinction risk or protection status influences a taxon’s price, a paired t-test analysis was conducted; t-tests ran for two forms of sale having an acceptable number of samples, i.e. for individual plants and individual seeds.

In order to record the Cretan endemic plants that can be considered as appealing to the tourism sector, we searched through the internet (September 2017) for agencies that organize botanical tours in Crete and we examined if they advertise any of the island’s endemic plants in their websites. With preliminary searches, we tried to identify the best possible keywords that would direct us to websites of interest (travel agencies, tour operators). We ended up with the following key phrases: ‘wildlife holidays + Crete’, ‘botanical tours + Crete’ and ‘botanical holidays + Crete’. We made Google searches using each of these phrases and recorded the relevant information included in the first 10 pages of the output (100 results), as these practically contained all information provided in the first 50 pages. We also recorded the country of origin of the agencies involved, time of the year when the tours take place and their duration, booking prices, practices that these companies follow and the plants mentioned in their websites, endemics or not. We assume that plants appearing in these webpages are highly attractive to the tourists targeted by these companies.

To be able to identify trends and patterns in e-trade and plant-oriented wildlife tourism, we used English for our study, as it is the most universal language for both international trade and tourism. An additional reason for using English was to have directly comparable results with a previous study regarding the Traded Endemic Plants of the Island of Cyprus [16]. Nevertheless, to have an estimate of how much different the results are if we use other languages, we made an additional study, at a later stage (2019), using French and German as the search languages in Google, for the traded taxa of the plant families of Lamiaceae and Liliaceae.

## Results

**Fig. 1:**
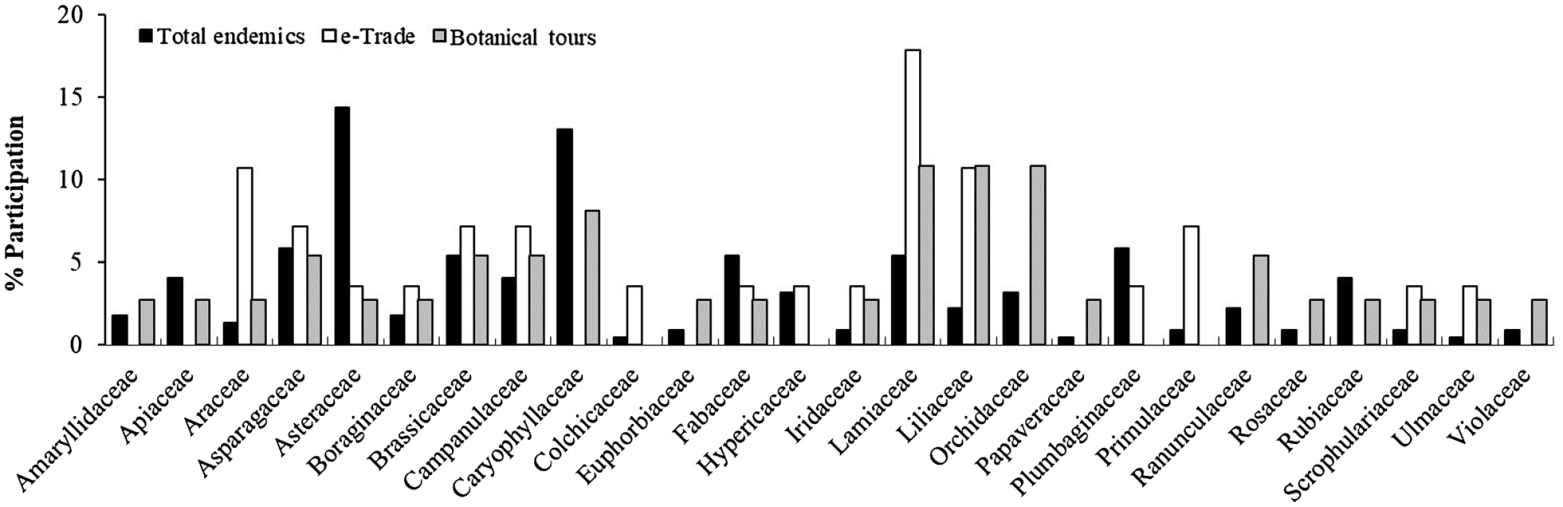
Frequency of occurrence (%) of plant families represented in the e-trade of the endemic plants of Crete, Greece (marked with white) and in wildlife tourism (marked with grey) and relative participation of these families in the endemic flora of Crete (marked with black)

The updated list of the local endemic plants of Crete that we produced includes 223 taxa (species and subspecies) (see Additional file 1). Of these, 28 taxa belonging to 16 families are traded via the internet (e-trade). Eight of these local endemics were found through the Royal Horticultural Society platform and six through the Plant Information Online webpage. Google search provided trade information for almost all (27) of these taxa. The Cretan endemics are traded by 65 nurseries from 14 countries (Table 1). No Asian or African countries are included among them (see Additional file 2). Lamiaceae is the family with the highest number of e-traded taxa; it is followed by Araceae and Liliaceae (Fig. 1).

**Table 1:** Summary results regarding the quantitative aspects for the electronic trade of the endemic taxa (species and subspecies) of Crete (Greece) and Cyprus [16]

Attributes	Quantity (% in parenthesis)	
	Crete	Cyprus
Endemic taxa	223	140
Endemic taxa traded (as percent of the island’s endemics)	28 (13)	24 (17)
Found through the Royal Horticulture Society Plant Finder	8	10
Found through Plant Information Online	6	3
Found through Google	27	22
Plant families represented	16	17
Countries involved	14	6
Nurseries involved	65	20
Nurseries selling each taxon	1–14	1–6
Taxa traded as living plants (as percent of the Traded Endemics of the Island [TEC])	17 (61)	10 (42)
Taxa traded as seeds (as percent of TEC)	14 (50)	20 (83)
Taxa traded as bulbs (as percent of TEC)	8 (29)	4 (17)
Taxa facing extinction risk (all relevant categories, as percent of TEC)	11 (39)	20 (83)
Taxa protected (all relevant categories, as percent of TEC)	14 (50)	12 (50)

Table 1 summarizes quantitative aspects of the e-trade of the endemic taxa of Crete. The most frequently encountered taxon is *Tulipa bakeri* traded by 14 nurseries, whereas the least frequently are *Arum idaeum*, *Biarum tenuifolium* subsp. *idomenaeum*, *Colchicum cretense*, *Fritillaria messanensis* subsp. *sphaciotica*, each traded by only one nursery. The genera *Biarum, Cyclamen*, *Origanum* and *Tulipa* have more than one representative in the list of traded Cretan endemics (see Additional file 3).

The most common form of sale is that of the living plant (17 taxa). Seeds and bulbs follow (14 and 8 taxa, respectively). Prices per living plant range from 2.58 € (2.95 $) on average for *Campanula cretica* to 36.75 € (42.26 $) on average for the only tree of the list, *Zelkova abelicea* (Table 2). Seed prices are distinguished per seed packet (containing undefined number of seeds), per gram, and per seed; for the latter form, they range from 0.02 € (0.02 $) for *Calamintha cretica* to 0.52 € (0.6 $) for *Crocus oreocreticus* and *Fritillaria messanensis* subsp*. sphaciotica.* Bulb prices range from 0.44 € (0.51 $) on average for *Tulipa bakeri* to 18 € (20.7 $) for *Colchicum cretense* (Table 2). In some cases, the range of prices for the same taxon and form of sale is large; at least more than double, it is for *Anchusa cespitosa*, *Cyclamen confusum, Erysimum mutabile* and *Origanum dictamnus*, for the living plant, and for *Crocus oreocreticus* and *Tulipa bakeri*, for the bulb (see Additional file 4).

**Table 2:** Average prices ± standard error in Euros (US Dollars, in parenthesis) of the Cretan endemics that are traded via the Internet as living plants, bulbs and/or seeds

Taxa	Prices, in € (in $)				
	Living plant	Individual bulb	Individual seed	Seed pack	Seeds per gram
1. *Acantholimon androsaceum* (Jaub. & Spach) Boiss.	3.45 ± 0.02 (3.97 ± 0.02)				
2. *Anchusa cespitosa* Lam.	11.16 ± 5.45 (12.8 ± 6.26)				
3. *Arum idaeum* Coustur. & Gand.	12.00 (13.72)				
4. *Bellevalia brevipedicellata* Turrill		8.18 ± 2.11 (9.40 ± 2.43)			
5. *Biarum davisii* Turrill	7.78 ± 0.79 (8.95 ± 0.91)	11.43 (13.1)			
6. *B. tenuifolium* subsp*. idomenaeum* P. C. Boyce & Athanasiou		15.99 (18.29)			
7. *Calamintha cretica* (L.) Lam	3.00 (3.45)		0.02 (0.02)	4.99 (5.74)	
8. *Campanula cretica* (A. DC.) D. Dietr.	2.58 (2.95)			3.42 ± 0.58 (3.93 ± 0.67)	
9. *Colchicum cretense* Greuter		18.00 (20.7)			
10. *Crocus oreocreticus* B. L. Burtt		2.38 ± 1.12 (2.73 ± 1.29)	0.52 (0.60)		
11. *Cyclamen confusum* (Grey-Wilson) Culham & al.	7.21 ± 1.21 (8.29 ± 1.39)		0.24 (0.28)		
12. *Cyclamen graecum* subsp*. candicum* Ietsw.ex Grey-Wilson	8.38 ± 2.10 (9.64 ± 2.41)		0.30 (0.35)		
13. *Draba cretica* Boiss. & Heldr.			0.22 (0.25)		45.00 (51.75)
14. *Ebenus cretica* L.			0.35 (0.40)	14.9 (17.14)	
15. *Erysimum mutabile* Boiss. & Heldr.	6.18 ± 0.82 (7.11 ± 0.94)				
16. *Fritillaria messanensis* subsp*. sphaciotica* (Gand.) Kamari & Phitos			0.52 (0.60)		
17. *Helichrysum heldreichii* Boiss.	8.72 ± 0.52 (10.03 ± 0.60)				
18. *Hypericum trichocaulon* Boiss. & Heldr. (probably *H. kelleri* Bald. in UK nurseries)	4.57 (5.22)			4.00 (4.60)	
19. *Muscari spreitzenhoferi* (Heldr. ex Osterm.) H. R. Wehrh.		8.2 ± 1.19 (9.42 ± 1.37)			
20. *Origanum dictamnus* L.	5.36 ± 0.79 (6.16 ± 0.91)				
21. *Origanum microphyllum* (Benth.) Vogel	3.74 ± 0.26 (4.30 ± 0.30)				
22. *Petromarula pinnata* (L.) A. DC.	8.47 ± 1.53 (9.74 ± 1.76)		0.1 ± 0.03 (0.12 ± 0.03)	2.50 (2.88)	
23. *Phlomis lanata* Willd.	7.25 ± 0.64 (8.33 ± 0.74)				
24. *Sideritis syriaca* L. subsp*. syriaca*				5.72 ± 2.27 (6.58 ± 2.61)	0.07 (0.08)
25. *Tulipa bakeri* A. D. Hall		0.44 ± 0.14 (0.51 ± 0.16)		4.31 ± 2.4 (4.95 ± 2.76)	
26. *Tulipa cretica* Boiss. & Heldr.		4.06 ± 0.33 (4.67 ± 0.38)			
27. *Verbascum arcturus* L.	6.93 (7.95)		0.08 ± 0.05 (0.09 ± 0.06)	5.59 ± 2.4 (6.43 ± 2.76)	
28. *Zelkova abelicea* (Lam.) Boiss.	36.75 ± 3.14 (42.26 ± 3.61)				

The exchange rates are as of July 17, 2017 (1 € = 1.15 $)

**Fig. 2:**
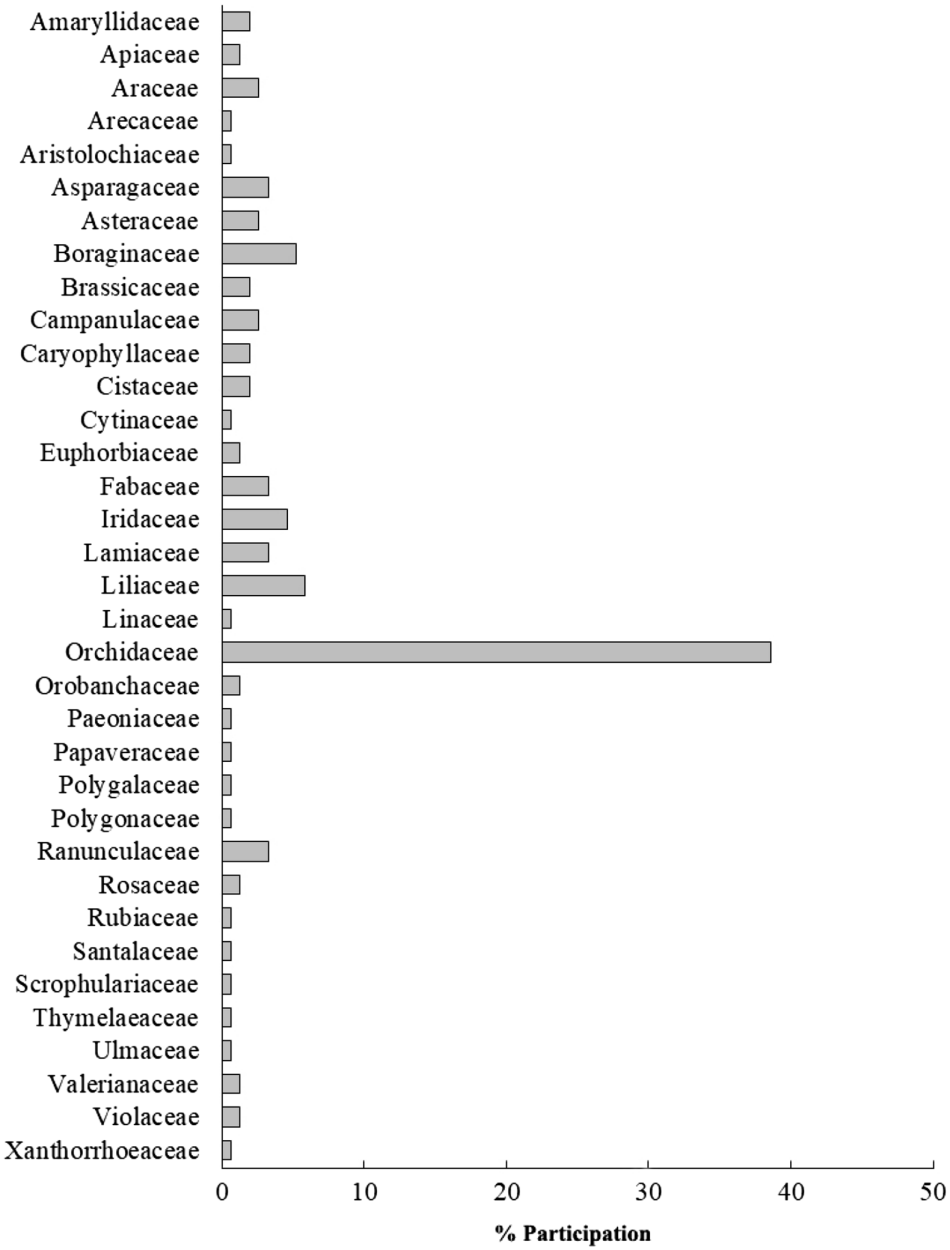
Frequency of occurrence (%) of plant families represented in the webpages of agencies offering botanical tours in Crete, Greece; all members of the flora of Crete are taken into consideration

We found 19 travel agencies advertising 153 taxa of the Cretan flora, endemic or not, in their webpages (Table 3). Orchidaceae is by far the prominent family with its representatives making 38.6% of the above taxa. All other families have a much lower representation, below 10% (Fig. 2). This striking difference does not hold true when members only of the endemic flora are taken into consideration. In this case, Orchidaceae, Lamiaceae and Liliaceae are all similarly represented (Fig. 1). In total, 37 endemic taxa belonging to 22 families were found in the webpages of 9 agencies (Table 3) (Additional file 5). Among these taxa, more than 40% are geophytes and *Tulipa doerfleri* is the most often advertised of all taxa (see Additional file 3).

**Table 3:** Summary results regarding the quantitative aspects of wildlife tourism focusing on the Cretan flora (data collected in September 2017)

Attributes	Quantity (% in parenthesis)
Agencies advertising plants of Crete	19
Plant taxa in the webpages of the agencies involved	153
Tour duration (days)	7 to 10
Tour cost per person in EUR, €; in USD, $	926 to 2715 €; 1150 to 3372 $
Endemic taxa in the webpages (percent of Cretan endemics)	37 (16.5)
Agencies advertising these endemic taxa	9
Plant families in which these endemic taxa belong	22
Endemic taxa facing extinction risk	13
Endemic taxa protected	20

**Fig. 3:**
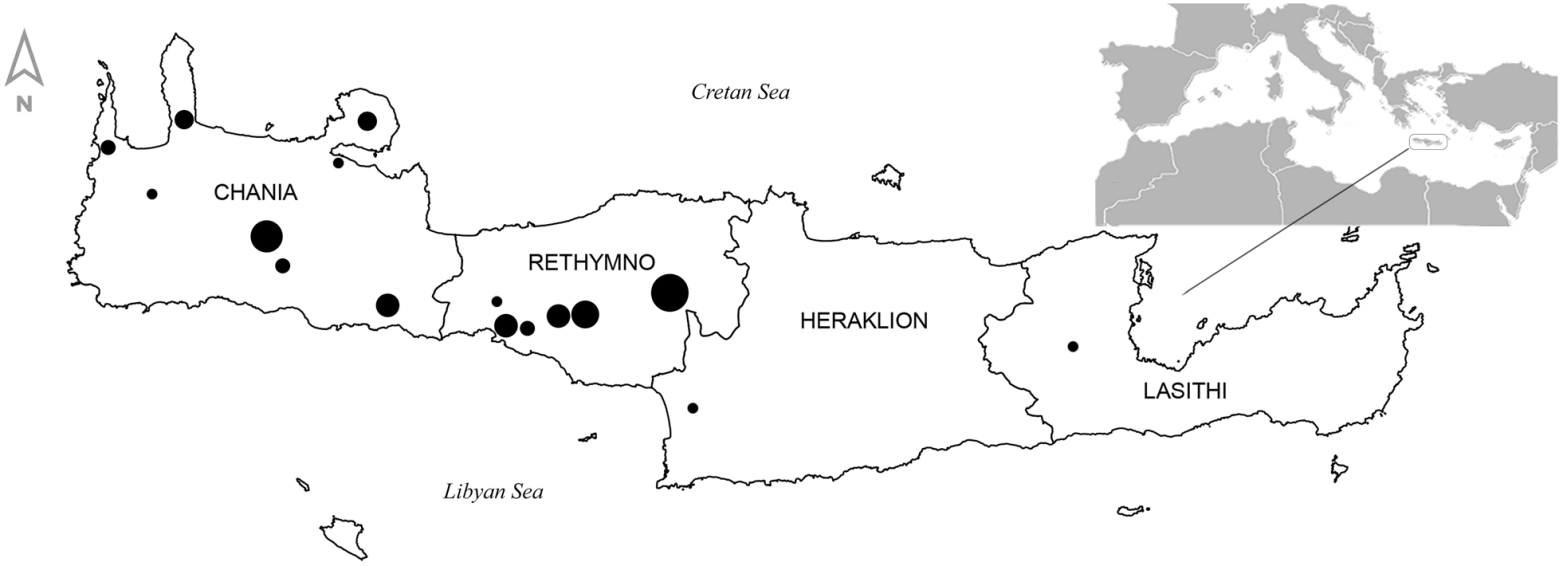
Areas visited in Crete, Greece, during botanical tours. The size of bullets is proportional to the number of endemic taxa (species and subspecies) advertised to be seen in each area; lowest value = 1, highest value = 9. Shown are also the four prefectures (Chania, Rethymno, Heraklion, Lasithi), in which the island is administratively divided and the position of Crete in the Mediterranean (inset)

The agencies offering tours of botanical character and advertising the local endemic plants of Crete are seated in Greece (three in Crete and one in Athens) and the United Kingdom (five). Wildlife holidays and plant-hunting tours are offered primarily in autumn and spring. They last seven to 10 days with a cost per person of the range 1000–3000 € (2017) (Table 3). Most of the travel agencies provide information on plant species that are to be encountered during the tour. They give the scientific and/or common names as well as the locations where these plants will be encountered. This information is presented either in the agencies’ websites and/or in the material produced after the end of the trip. Tours are usually guided by well-trained personnel. They cover the whole island of Crete but they are mainly concentrated in the prefectures of Rethymnon and Chania, where the two major Cretan mountain massifs are located (Fig. 3). Gorges, plateaus, coastal areas and mountain-tops are the most visited types of habitat.

The overall number of taxa detected in association with e-trade and wildlife tourism is 54; in both activities involved are only 12 taxa corresponding to 22.6% of the total number. Taxa facing extinction risk [50–54] or protected under international frameworks [EU Habitats Directive (92/43/EEC), CITES, Bern Convention)] that Greece has either incorporated in its national legislation [Common Ministerial Agreement 33318/3028/11-12-1998 (Governmental Gazette 1289/Β/28-12-98) as amended by the Common Ministerial Agreement 14849/853/Ε103/4-4-2008 (Governmental Gazette 645/Β/11-4-08)] and similarly others that are protected at national level [Presidential Decree 67/81 (Governmental Gazette 23/Α/81) for the protection of native flora and wild fauna] are included in both trade and tourism activities (Table 4).

**Table 4:** Cretan endemics involved in the e-trade and wildlife tourism that are protected under different frameworks and/or face extinction risk

Protection framework and risk assessment	Endemic taxa in e-trade	Endemic taxa in wildlife tourism
Endangered	^*3*^*Bellevalia brevipedicellata*, ^*1*^*Zelkova abelicea*	^*2*^*Cephalanthera cucullata*, ^*2*^*Orchis sitiaca*, ^*1*^*Zelkova abelicea*
Vulnerable	^*5*^ *Acantholimon androsaceum*	^*3*^*Dianthus xylorrhizus,*^*4*^*Tulipa doerfleri*, ^*4*^*Ranunculus cupreus*
Near threatened	^*2*^*Biarum davisii*, ^*3*^*Helichrysum heldreichii*, ^*2*^*Origanum dictamnus*	^*3*^*Euphorbia sultan*-*hassei*, ^*2*^*Origanum dictamnus*
Rare	^*4*^*Anchusa cespitosa*, ^*4*^*Biarum tenuifolium* subsp*. idomenaeum*, ^*4*^*Campanula cretica*, ^*4*^*Crocus oreocreticus*, ^*4*^*Calamintha cretica*	^*4*^*Anchusa cespitosa*, ^*4*^*Corydalis uniflora*, ^*4*^*Dianthus fruticosus* subsp. *creticus*, ^*4*^*Sanguisorba cretica,* ^4^*Teucrium alpestre*
Directive 92/43/EEC	*Origanum dictamnus*, *Zelkova abelicea*	*Origanum dictamnus*, *Cephalanthera cucculata*, *Zelkova abelicea*
CITES	*Cyclamen graecum* subsp*. candicum*, *C. confusum*	
Bern Convention		*Origanum dictamnus*, *Cephalanthera cucullata*, *Zelkova abelicea*
Greek Presidential Decree 67/1981	*Anchusa cespitosa*, *Bellevalia brevipedicellata*, *Biarum davisii*, *B. tenuifolium* subsp. *idomenaeum*, *Calamintha cretica*, *Campanula cretica*, *Crocus oreocreticus*, *Helichrysum heldreichii*, *Origanum dictamnus*, *Phlomis lanata*, *Tulipa bakeri*, *T. cretica*, *Zelkova abelicea*	*Allium circinnatum*, *Anacamptis papilionacea* subsp*. alibertis*, *Anchusa cespitosa*, *Cephalanthera cucullata*, *Corydalis uniflora*, *Dianthus fruticosus*, *D. xylorrhizus*, *Ferulago thyrsiflora*, *Himantoglossum samariense*, *Orchis sitiaca*, *Origanum dictamnus*, *Phlomis lanata*, *Ranunculus cupreus*, *Sanguisorba cretica*, *Scilla nana*, *Tulipa bakeri*, *T. cretica*, *T. doerfleri*, *Viola alba* subsp. *cretica*, *Zelkova abelicea*

The sources for current (Endangered, Vulnerable, Near Threatened) and older (Rare) IUCN extinction risk assignments are as follows: 1 = [55], 2 = [54], 3 = [53], 4 = [52], 5 = [51]

Overall, 13 endangered, vulnerable, near threatened, rare and/or internationally protected taxa are involved in the electronic trade (Table 1); this number rises to 16 if those protected at national level are also included. Similarly, 12 taxa facing extinction risk and/or protected at international level are involved in tourism activities; the number rises to 22 if taxa protected at national level are also included (Tables 3, 4). Paired t-test analyses showed no significant (*p* > 0.05) price difference between the group of taxa facing extinction risk or being protected and the group of other taxa for the two forms of sale examined (Table 5).

**Table 5:** Average prices in Euros ± standard error for two forms of sale of the endemic plants of Crete, Greece, and results of t-test analysis

Form of sale	Group of taxa I		Group of taxa II		*t*-value	*p*-value
	Average price (± se)	N	Average price (± se)	N		
Living plant	9.23 (± 2.87)	11	6.99 (± 1.21)	6	0.560	0.583
Seed	0.27 (± 0.10)	4	0.25 (± 0.08)	5	0.123	0.905

Plants are divided in two groups consisting of (i) taxa facing extinction risk and/or being under protection (Group I) and (ii) all other taxa (Group II); N = number of samples

The additional search that we did for the families of Lamiaceae and Liliaceae in German and French, at a later stage (2019 against 2016–2017, when the bulk of the search was conducted), did not add any new species to the list of those already found neither for Lamiaceae nor for Liliaceae. But *Phlomis lanata* was missing from the traded taxa. As was the case with the search in English, *Tulipa bakeri* was the taxon sold from most nurseries and the UK was the country represented by most vendors. However, there was a dramatic increase in the number of vendors involved: 66 new ones were added. These originated from countries where German and French are spoken, such as Germany (22), Austria (2), Switzerland (2), and France (12), but also from other countries including the English-speaking UK (13), USA (8) and Australia (1). With this additional search, another four countries (Austria, Canada, Israel and Sweden) were identified as vendors’ origin but four of those previously detected were now missing (Greece, Italy, New Zealand and Spain). These results were not assessed further and are not included in figures and tables.

## Discussion

### Attractiveness of the Cretan endemic plants

The relative representation of plant families in the e-trade and wildlife tourism involving the endemic plants of Crete diverges in several cases from their relative participation to the island’s endemic flora (Fig. 1). In both markets, the family of Lamiaceae ranks first corresponding to 17.9% of the traded and 11% of the advertised taxa (in the latter case, equally represented with Liliaceae and Orchidaceae), when it makes only 5.4% of the Cretan endemic flora. Such disproportionately high participation in the e-trade is observed also for Araceae, Liliaceae, Primulaceae, and for Orchidaceae in wildlife tourism. In contrast, Asteraceae, which is the most widely represented family among the Cretan endemics, is of low occurrence in both markets. Interestingly, families of high ornamental value like Caryophyllaceae or Orchidaceae do not feature in e-trade; the absence of the latter family from e-trade could be attributed to the high sensitivity of its members in cultivation.

Overall, the values embedded in the plants participating in the e-trade are of ornamental, aromatic, medicinal, and functional, in general, character. Notable examples of the e-traded endemic plants that represent these values are *Tulipa bakeri*, *Zelkova abelicea*, and *Sideritis syriaca* subsp*. syriaca.* The ornamental *T. bakeri* (lilac wonder), with rare for tulips pink flowers, is the most commonly encountered endemic plant of Crete in nurseries around the world, with its bulbs having the highest price for this form of sale. This outstanding tulip participation in the e-trade brings to mind the tulip mania of the 17th century in Holland, when tulips, after their introduction there, became so expensive that they were even used as money, till the market in them crashed offering one of the first examples of economic bubbles in history [56]. *Zelkova abelicea* has the highest price in the form of living plant. This endemic species is a representative of a tree genus of the Tertiary. Its wood was traditionally used by the local people for producing walking sticks (‘katsounes’, in the local dialect) by bending its flexible young shoots [57]. *Sideritis syriaca* subsp*. syriaca* is an aromatic plant of low essential oil content [58] that has been traditionally used as a tea against colds and sore throats, stomach and respiratory illnesses. Its antimicrobial, anti-inflammatory and analgesic properties were verified and other important medicinal qualities like hypotensive, vasorelaxant, cardiodepressant, anti-oxidant and anti-tumor were recently found in representatives of the genus *Sideritis* [59, 60] increasing the demand for the plant. Due to severe population decline during the last decades, regulations have been imposed by the local forest service regarding collection and trade of this plant, and a pilot project has run at the Mediterranean Agronomic Institute of Chania, Crete [61], in cooperation with the Forest Directorate of Chania for the long-term monitoring and evaluation of its wild populations. Results of this project focusing on the important aromatic-medicinal plants of Crete showed that only in the region of Chania 56 farms cultivate such plants, among which the two Cretan endemics of high demand *Origanum dictamnus* and *Sideritis syriaca* subsp. *syriaca* (pers. com., according to E. Stamataki). Restrictions and regulations of collection from the wild in combination with the high demand of these plants led to a new activity of increasing importance in Crete, that of cultivation of local herbs. Conducted in an integrated way, this activity that results into certified products can reduce the pressure on the plants’ natural populations. It can also strengthen the local communities by bringing profits to them and allowing them to share the benefits deriving from the development and use of the local resources.

Our results suggest that the protection status or extinction risk does not play a major role with respect to the prices that these plants attain in the market (Table 5). For instance, *Origanum dictamnus* that is included in Directive 92/43/EEC is sold as living plant at a price much lower than the average after all traded taxa. This implies that it can be rather easily acquired, which is the case when plants are cultivated and/or when their propagation and growth do not require special treatments and concomitant costs. *Origanum dictamnus* is indeed under cultivation in Crete [62, 63]. Similarly, under cultivation but only to a small scale on the island is *Sideritis syriaca* subsp*. syriaca* and *O. microphyllum* [pers. com., according to E. Stamataki]. We could not find information or indications of cultivation for commercial purposes for any other of the Cretan endemics. To examine whether the result would differ if we excluded the widely cultivated *O. dictamnus* from the group of protected and/or facing extinction risk taxa, we rerun the analysis without it. Again, prices did not differ. For some taxa, the range of prices for the same form of sale is large. For instance, for *O. dictamnus* and the form of living plant, the lowest selling price is 2.84 € (3.25 $) whereas the highest is 8 € (9.2 $). It is not known if the considerably higher prices for the same plant material correspond to a superior value associated with wild origin or if they simply reflect plant size in pots, transport costs, the company’s stock or differences in supply and demand at the time.

Exploring their webpages, we found that most of the vendors did not provide information on the origin of the plants that they were selling. We also found some who advertised the wild source of this plant material they were selling without providing any authorization for this (e.g. hillviewrareplants.com.au). It is not known how much pressure is put on the wild populations of the traded taxa, but the situation for some of them may become very serious [15, 16].

The additional search that we did in 2019 for the families of Lamiaceae and Liliaceae provided results showing not only more vendors from the countries where French and German are spoken but also from the English-speaking world. In fact, nurseries from the latter countries made a third of the new ones. Although four more countries were identified as vendors’ origin, another four were missing from the new list including Greece. The fact that the nurseries from the English speaking countries were not detected before with the search in English suggests that the e-trade in Cretan endemics increases at a very high rate.

### Comparison of patterns in electronic trade for Crete and Cyprus

Given the existence of e-trade data for the endemics of Cyprus [16], the largest island of east Mediterranean, and the similarities between Crete and Cyprus in geographical, environmental [1] and political aspects (both making part of the European Union), we examined how similar are the results regarding the e-trade of their local endemic plants. Table 1 summarizes quantitative aspects of the e-trade of the endemic taxa of Cyprus in comparison to the respective data for Crete.

Like Crete, Cyprus is a biodiversity hotspot of the Eastern Mediterranean. The two islands are of similar size, 8400 km^2^ and 9251 km^2^, respectively, and of similar topography: both have high mountains, up to 2456 m in Crete (Mt Ida or Psiloritis), and up to 1952 m in Cyprus (Mt Olympus; of same name with the highest mountain of Greece). Crete has an endemic flora 1.6 times richer than Cyprus. This difference is not reflected in the number of traded taxa that are rather similar, 28 and 24, respectively. Geophytes make about half (45%) of the traded Cretan endemics (see Additional file 3), whereas only 21% of the Cypriot ones [16]. The family of Lamiaceae also ranks first among the traded endemic plants of Cyprus with almost identical contribution (17–18%) but nine families with endemic taxa occurring in both islands have traded representatives from only one. This holds true for Campanulaceae, Hypericaceae, Liliaceae, Plumbaginaceae, Caprifoliaceae, Caryophyllaceae, Crassulaceae, Euphorbiaceae and Orchidaceae. Representatives of the first four families appear among the e-traded taxa only of Crete, whereas of the latter five among the e-traded taxa only of Cyprus [16] (see Additional file 3). Of these nine families, Liliaceae are represented with three taxa, Crassulaceae and Campanulaceae with two, whereas the remaining six with one traded taxon. Geophytes and members of the mint family make the great bulk of the traded endemic taxa of Crete (64%) but not so in the case of Cyprus, where the market seems to be more diversified.

There are remarkably more vendors trading Cretan endemic plants than Cypriot ones [16], 65 vs. 20. United Kingdom is the first in rank country-origin of the vendors and similarly USA the second for both islands, but with quite different share of the whole market. Of the vendors involved in e-trade, those from the UK make 38% for the Cretan endemics, whereas 66% for the Cypriot ones. For vendors from the USA, the contribution is reversed: it is 31% and 15%, respectively. In the case of Cretan endemics, the other 12 countries have only minor contribution (1.4–6.8%), but included among these countries are very distant ones like Australia and New Zealand. For most of the countries with nurseries selling them, the trading activity reflects their long horticultural tradition and/or the robustness of their ornamental plant industry [15]. Presence of Greek communities in some of the countries with nurseries selling the Cretan endemics may also have played a role in the demand for these plants. In the case of Cypriot endemics, the overwhelming contribution of companies from the UK can be associated with historical reasons, as the island was under the British rule for a considerable part of the 19th and 20th centuries.

To a certain extent, the preponderance of vendors from English speaking countries for both islands may be related with the fact that searches were made in English. Even so, within the English speaking world, there is a clear difference between vendors of Greek and Cypriot endemics.

### Comparison of patterns in electronic trade and wildlife tourism

There is not much published information on tourism targeting the wildlife of Greece. Actually, this is the first time that such a study has been conducted regarding this country’s endemic flora. To our knowledge, the same holds true in a wider context, for other countries as well. In this study, we focused on tourists who book specialized, plant-oriented holidays that include in situ observations of Cretan plants, not on those that take excursions of general character that include aspects of nature, mostly lasting a single day. These tourists are usually willing to pay a substantial amount of money in order to admire and capture the representatives of the Cretan flora in their natural environment. Extinction risk and/or protection status of the Cretan endemics may play a role for inclusion of such plants as attractions in the organized tours, but not the major one: plants of this type make 35% of the advertised flora in the agencies’ webpages. Taking as a criterion of a taxon’s attractiveness the number of agencies referring to it, it seems that the endemic plants of Crete that are the most traded are not the most attractive to tourists. The two markets are similar in that geophytes play a prominent role with comparable participation in their activities (40–45% representation) and so do Lamiaceae that rank first among the represented plant families. Nevertheless, only 12 taxa are common to both, corresponding to 22.6% of all traded and tourism related Cretan endemic taxa (see Additional file 2). To a certain extent, this could be due to the blooming times of the plants, which do not fall within the most convenient periods of the year for tourists from the point of view of weather and major flowering season.

Our assessment showed that companies from the UK are very active not only in e-trade but also in plant-oriented wildlife tourism. Half of the tour operators offering holidays of botanical interest in Crete are located in the UK. Given that our searches were conducted in English, these results may be considered as language-biased. Nevertheless, they are indicative of species and areas, currently of high interest in wildlife tourism.

The use of plants’ scientific names in the webpages of agencies involved in this type of tourism indicates that the audience targeted is educated with a special interest in nature. Professionals involved in this activity are very explicit in showing their concern about the environmental footprint of the trip, their interest in sustainable tourism and their intention to respect the local culture and help the local economy. A number of companies inform their potential clients on the legislation forbidding collecting, eradication or trading of the wild flora and ask them to respect it. Others ask from their clients not to give detailed information on the sites where these plants occur or advise them not to use GPS during the tour due to the rarity of some plants that are to be encountered—although a simple photo taken with a smartphone may be easily georeferenced.

This obedience to legislation and rules exhibited by agencies involved in wildlife tourism is not similarly expressed by nurseries involved in the e-trade of the Cretan endemics. While searching for the practices that the latter follow, we found nursery holders openly admitting that they travel to Crete in order to collect propagation material from wild populations (webpages available from the authors upon request) and satisfy their clients. This violates the Greek law of biodiversity requiring special permits for collection, the Nagoya protocol on access to genetic resources, which Greece has signed and ratified, and the related Regulation of the European Union (EU Regulation 511/2014).

Plant-oriented tourism is a growing business on the island of Crete. For the moment, the involved tourist population is of rather small size and consists of plant admirers who enjoy observing plants in their natural habitats. Given this and the seemingly responsible attitude of tour operators, as evidenced in their information material, this activity does not seem currently to impact seriously the endemic flora of the island. Electronic trade of genetic resources, in contrast, is a big market difficult to control. Most nurseries provide little or no information on the sources of the plants they sell, while others, as mentioned above, advertise collection from the wild without holding licenses. In fact, it is not clear how much of the traded material originates from the wild and how much this activity conforms to existing rules. Despite some similarities, the two markets seem independent of each other in many aspects.

### Conservation implications

According to the Greek law for the protection of biodiversity [3937/11 (Governmental Gazette 60/Α/31-3-2011)], collection and trade of endemic plants is forbidden. They are only allowed when these plants are important for the local production and consumption and only when other Greek or European legislation or specific action plans for these plants do not forbid these activities. The forest services of the country are responsible for issuing permits for plant collection. Permits should be issued also for scientific research of these plants in their natural environment. The Forest Service of the Prefecture of Chania in Crete [64] regulates the collection of several plants, among which of the endemics *Sideritis syriaca* subsp*. syriaca* (Cretan mountain tea, malotira) and *Origanum microphyllum* (Cretan marjoram). For a small quantity for personal use, there is no need for a special permit. For other purposes, interested parties should require and get such permits, which define the area where to collect, the maximum amount allowed, and the collection methods. Interestingly, the last phrase of this year’s (2019) decision of the Forest Service of Chania is the following: ‘We commission the implementation of this provision to the bodies of the Forest Service, the Police and the Hunting Guards, and to law-abiding and forward-looking citizens’. The provision of the law for collection of plants from the wild for local production and consumption makes local forest services responsible for evaluating the situation in the areas of their jurisdiction and impose restrictions on plant collection, when needed, so that the natural populations do not decline. It also allows local communities to benefit from the ecosystem services of their natural environment and by doing so it enables local people to take responsibility for the sustainable use of the wild growing endemic plants and other local resources. This is not the case with international e-trade; plant traders do not show such a responsibility and income generation is outside the region.

Our results suggest that priority in plant monitoring schemes in Crete should be given to single-island endemic plants that participate in the e-trade, particularly on (i) those of highest demand, and (ii) those protected and/or facing extinction risk. Taking as a criterion of demand the number of nurseries selling each taxon, for (i), this is primarily the case of *Erysimum mutabile, Origanum dictamnus*, *Phlomis lanata* and *Tulipa bakeri* that appear in approximately 10% or more of the 65 nurseries selling endemic plants of Crete. However, as *Phlomis lanata* is a common component of the extended scrub communities in the central and eastern parts of the island [30], it should not be included in the list of priority species. For (ii), this is the case of endangered, vulnerable, near threatened and rare taxa, after IUCN extinction risk assessments, almost all of which are also protected, i.e. *Acantholimon androsaceum*, *Anchusa cespitosa*, *Bellevalia brevipedicellata*, *Biarum davisii*, *B. tenuifolium* subsp. *idomenaeum*, *Calamintha cretica*, *Campanula cretica*, *Crocus oreocreticus*, *Cyclamen confusum*, *C. graecum* subsp. *candicum*, *Helichrysum heldreichii*, *Origanum dictamnus* and *Zelkova abelicea.*

Although plant-oriented wildlife tourism does not seem to have currently major impacts, taxa and their habitats that are very frequently visited must be taken into consideration when planning monitoring schemes. This is the case of *Tulipa doerfleri* and *Petromarula pinnata* that feature in the webpages of at least 50% of the agencies involved. Whereas *T. doerferi* is restricted to a single mountain plateau and, hence, requiring special attention, *P. pinnata* is a rock-dwelling Cretan endemic, quite common on the island; therefore, it should not be included among the priority species.

As a result, the list of the Cretan endemic plants requiring special attention includes 16 of the 18 aforementioned taxa. Monitoring activities should be accompanied by controls by the competent authorities to make sure that there is no illegal collection of the country’s genetic resources and no unauthorized exportation. Training of the personnel of these authorities to exert this role is very important as botanical knowledge and flora protection were not till recently among their usual duties and area of expertise. Monitoring, regular controls and efficient custom supervision are practices that the relevant local authorities should follow seriously and consistently so as to protect the unique flora of this biodiversity hotspot.

## Conclusions

The endemic taxa of Crete have a considerable involvement in electronic trade and wildlife tourism, with e-trade increasing at a very high rate. Geophytes and members of the mint family dominate the two markets. They also dominate the e-traded endemic plants of Cyprus implying a high demand for ornamental and aromatic/medicinal plants from east-Mediterranean islands. Although most of the tourists may have a genuine interest in the unique floristic elements of the island, some may also have disguised commercial interests. Nevertheless, plant-oriented wildlife tourism has not gone beyond the involvement stage of the tourist destination life-cycle [65] and, hence, it is not expected to have major negative impacts to taxa and habitats in the immediate future. However, more targeted studies should explore the rate of increase of this type of tourism, the specific interests of the participating tourists and the behavior in the field of tourists and professionals alike. Current reality is different with e-trade: how much this activity conforms to rules is not clear nor is it known how much of the traded material originates from the wild. Based, on the one hand, on the high frequency of occurrence of the Cretan endemics in nurseries around the globe and the information material of travel agencies and, on the other, on IUCN extinction risk assessments and protection status, 16 of the 223 Cretan endemics were identified as priority species in need of special attention. These are: *Acantholimon androsaceum*, *Anchusa cespitosa*, *Bellevalia brevipedicellata*, *Biarum davisii*, *B. tenuifolium* subsp. *idomenaeum, Calamintha cretica*, *Campanula cretica*, *Crocus oreocreticus, Cyclamen confusum*, *C. graecum* subsp. *candicum*, *Erysimum mutabile*, *Helichrysum heldreichii*, *Origanum dictamnus*, *Tulipa bakeri*, *T. doerfleri* and *Zelkova abelicea.* Monitoring, regular controls and efficient custom supervision are practices that the relevant authorities should follow to protect the unique flora of the island.

## Supplementary information

**Additional file 1.** Checklist of endemic species and subspecies in Crete.

**Additional file 2.** Electronic addresses of the nurseries involved in the trade of Cretan endemic plants and countries where these are located.

**Additional file 3.** Endemic taxa of Crete that are traded by nurseries via the internet or advertised by tourist agencies to attract visitors.

**Additional file 4.** Prices in Euros (US Dollars, in parenthesis) of the Cretan endemic taxa that are traded by nurseries via the Internet as living plants, bulbs and seeds. The exchange rate for Euros and Dollars are as of July 17, 2017 (1 € = 1.15 **$**).

**Additional file 5.** Electronic addresses of tourist agencies using Cretan endemic plants to attract tourists, and countries where these are located.

## Data Availability

The datasets generated and/or analyzed during the current study are available from the corresponding author on reasonable request.

## Abbreviation

TEC: Traded Endemic plants of Crete
