# Supplementary material for: Endemic plants of Crete in electronic trade and wildlife tourism: current patterns and implications for conservation
Source: J Biol Res (Thessalon). 2019 Oct 30;26:10. doi: 10.1186/s40709-019-0104-z (PMC6822446; doi:10.1186/s40709-019-0104-z)
Supplement: Supplementary file 1 — Additional file 1. Checklist of endemic species and subspecies in Crete. [file 40709_2019_104_MOESM1_ESM.docx]

**Additional file 1.** Checklist of endemic species and subspecies in Crete

**Pteridophytes**

**Aspleniaceae**

*Asplenium creticum* Lovis & al.

**angiosperms**

**Amaryllidaceae**

*Allium bourgeaui* subsp. *creticum* Bothmer

*Allium circinnatum* Sieber subsp. *circinnatum*

*Allium dilatatum* Zahar.

*Allium platakisii* Tzanoud. & Kypr.

**Apiaceae**

*Bupleurum gaudianum* Snogerup

*Bupleurum kakiskalae* Greuter

*Chaerophyllum creticum* Boiss. & Heldr.

*Eryngium ternatum* Poir.

*Ferulago thyrsiflora* (Sm.) W. D. J. Koch

*Geocaryum creticum* (Boiss. & Heldr.) Engstrand

*Horstrissea dolinicola* Greuter & al.

**Araceae**

*Arum idaeum* Coustur. & Gand.

*Biarum davisii* Turrill

*Biarum tenuifolium* subsp. *idomenaeum* P. C. Boyce & Athanasiou

**Asclepiadaceae**

*Vincetoxicum creticum* Browicz

**Asparagaceae**

*Bellevalia brevipedicellata* Turrill

*Bellevalia juliana* Bareka & al.

*Bellevalia sitiaca* Kypr. & Tzanoud.

*Muscari spreitzenhoferi* (Heldr.) H. R. Wehrh.

*Ornithogalum dictaeum* Landström

*Ornithogalum insulare* Kypr. & al.

*Prospero battagliae* Speta

*Prospero depressum* Speta

*Prospero hierapytnense* Speta

*Prospero idaeum* Speta

*Prospero rhadamanthi* Speta

*Prospero talosii* (Tzanoud. & Kypr.) Speta

*Scilla nana* (Schult. & Schult. f.) Speta

*Scilla nana* subsp. *albescens* (Speta) Speta

*Scilla nana* subsp. *nana*

**Asteraceae**

*Anthemis abrotanifolia* (Willd.) Guss.

*Anthemis filicaulis* (Boiss. & Heldr.) Greuter

*Anthemis glaberrima* (Rech. f.) Greuter

*Anthemis samariensis* Turland

*Anthemis tomentella* Greuter

*Carlina diae* (Rech. f.) Meusel & Kästner

*Centaurea argentea* subsp. *chionantha* (Turland & L. Chilton) Greuter

*Centaurea argentea* subsp. *macrothysana* (Rech. f.) Turland & L. Chilton

*Centaurea baldaccii* Degen ex Halácsy

*Centaurea idaea* Boiss. & Heldr.

*Centaurea lancifolia* Sieber ex Spreng.

*Centaurea poculatoris* Greuter

*Centaurea redempta* Heldr. subsp. *redempta*

*Cirsium morinifolium* Boiss. & Heldr.

*Crepis auriculifolia* Sieber ex Spreng.

*Crepis sibthorpiana* Boiss. & Heldr.

*Filago wagenitziana* Bergmeier

*Helichrysum doerfleri* Rech. f.

*Helichrysum heldreichii* Boiss.

*Hieracium schmidtii* subsp. *creticum* (Zahn) Greuter

*Hypochaeris tenuiflora* (Boiss.) Boiss.

*Inula candida* subsp. *decalvans* (Halácsy) P. W. Ball ex Tutin

*Klasea cretica* (Turrill) Holub

*Lactuca alpestris* (Gand.) Rech. f.

*Onopordum bracteatum* subsp. *creticum* Franco

*Phagnalon pygmaeum* (Sieber) Greuter

*Scorzonera mollis* subsp. *idaea* (Gand.) Lack

*Senecio fruticulosus* Sm.

*Staehelina petiolata* (L.) Hilliard & B. L. Burtt

*Tragopogon lassithicus* Rech. f.

**Boraginaceae**

*Alkanna sieberi* DC.

*Anchusa cespitosa* Lam.

*Cynoglossum sphacioticum* Boiss. & Heldr.

*Myosotis solange* Greuter & Zaffran

**Brassicaceae**

*Alyssum fragillimum* (Bald.) Rech. f.

*Alyssum idaeum* Boiss. & Heldr.

*Alyssum lassiticum* Halácsy

*Alyssum sphacioticum* Boiss. & Heldr.

*Arabis cretica* Boiss. & Heldr.

*Draba cretica* Boiss. & Heldr.

*Erysimum creticum* Boiss. & Heldr.

*Erysimum mutabile* Boiss. & Heldr.

*Erysimum raulinii* Boiss.

*Noccaea cretica* (Degen & Jáv.) F. K. Mey.

*Noccaea zaffranii* F. K. Mey.

*Ricotia cretica* Boiss. & Heldr.

**Campanulaceae**

*Campanula cretica* (A. DC.) D. Dietr.

*Campanula creutzburgii* Greuter

*Campanula hierapetrae* Rech. f.

*Campanula jacquinii* (Sieber) A. DC.

*Campanula pelviformis* Lam.

*Campanula saxatilis* L. subsp. *saxatilis*

*Campanula spatulata* subsp. *filicaulis* (Halácsy) Phitos

*Campanula tubulosa* Lam.

*Petromarula pinnata* (L.) A. DC.

**Caryophyllaceae**

*Bolanthus creutzburgii* Greuter

*Bolanthus creutzburgii* subsp. *creutzburgii*

*Bolanthus creutzburgii* subsp. *zaffranii* Phitos & al.

*Bufonia stricta* subsp. *cecconiana* (Bald.) Rech. f.

*Cerastium brachypetalum* subsp. *doerfleri* (Halácsy ex Hayek) P. D. Sell & Whitehead

*Cerastium deschatresii* Greuter & al.

*Cerastium scaposum* Boiss. & Heldr.

*Cerastium scaposum* subsp. *peninsularum* Greuter & al.

*Cerastium scaposum* subsp. *scaposum*

*Cherleria wettsteinii* (Mattf.) A. J. Moore & Dillenb.

*Dianthus fruticosus* subsp. *creticus* (Tausch) Runemark

*Dianthus fruticosus* subsp. *sitiacus* Runemark

*Dianthus juniperinus* Sm.

*Dianthus juniperinus* subsp. *aciphyllus* (Sieber ex Ser.) Turland

*Dianthus juniperinus* subsp. *bauhinorum* (Greuter) Turland

*Dianthus juniperinus* subsp. *heldreichii* Greuter

*Dianthus* *juniperinus* subsp. *idaeus* Turland

*Dianthus* *juniperinus* subsp. *juniperinus*

*Dianthus* *juniperinus* subsp. *kavusicus* Turland

*Dianthus* *juniperinus* subsp. *pulviniformis* (Greuter) Turland

*Dianthus sphacioticus* Boiss. & Heldr.

*Dianthus xylorrhizus* Boiss. & Heldr.

*Petrorhagia candica* P. W. Ball & Heywood

*Petrorhagia dianthoides* (Sm.) P. W. Ball & Heywood

*Silene ammophila* Boiss. & Heldr. subsp. *ammophila*

*Silene antri-jovis* Greuter & Burdet

*Silene flavescens* subsp. *dictaea* (Rech. f.) Greuter

*Silene integripetala* subsp. *greuteri* (Phitos) Akeroyd

*Silene pinetorum* Boiss. & Heldr.

*Silene pinetorum* subsp. *pinetorum*

*Silene pinetorum* subsp. *sphaciotica* Oxelman & Greuter

*Silene sieberi* Fenzl

*Silene variegata* (Desf.) Boiss. & Heldr.

*Telephium imperati* subsp. *pauciflorum* (Greuter) Greuter & Burdet

**Colchicaceae**

*Colchicum cretense* Greuter

**Convolvulaceae**

*Convolvulus argyrothamnos* Greuter

*Cuscuta atrans* Feinbrun

**Crassulaceae**

*Sedum eriocarpum* subsp. *spathulifolium* ’t Hart

**Cyperaceae**

*Carex cretica* Gradst. & J. Kern

*Carex idaea* Greuter & al.

**Dipsacaceae**

*Lomelosia albocincta* (Greuter) Greuter & Burdet

*Lomelosia minoana* (P. H. Davis) Greuter & Burdet

*Lomelosia minoana* subsp. *asterusica* (Greuter) Greuter & Burdet

*Lomelosia minoana* subsp. *minoana*

*Lomelosia sphaciotica* (Roem. & Schult.) Greuter & Burdet

*Lomelosia sphaciotica* subsp. *decalvans* (Halácsy) Bergmeier

*Lomelosia sphaciotica* subsp. *sphaciotica*

**Euphorbiaceae**

*Euphorbia rechingeri* Greuter

*Euphorbia sultan-hassei* Strid & al.

**Fabaceae**

*Astragalus creticus* Lam.

*Astragalus creticus* subsp. *creticus*

*Astragalus creticus* subsp. *minoicus* Brullo & Giusso

*Astragalus dolinicola* (Brullo & Giusso) Brullo & Giusso

*Astragalus idaeus* Bunge

*Astragalus nummularius* Lam. subsp. *nummularius*

*Ebenus cretica* L.

*Lathyrus neurolobus* Boiss. & Heldr.

*Medicago arborea* subsp. *strasseri* (Greuter & al.) Sobr.-Vest. & Ceresuela

*Onobrychis sphaciotica* Greuter

*Ononis verae* Širj.

*Securigera globosa* (Lam.) Lassen

*Trifolium phitosianum* N. Böhling & al.

**Fumariaceae**

*Corydalis uniflora* (Sieber) Nyman

**Hypericaceae**

*Hypericum aciferum* (Greuter) N. Robson

*Hypericum amblycalyx* Coustur. & Gand.

*Hypericum empetrifolium* subsp. *tortuosum* (Rech. f.) I. Hagemann

*Hypericum jovis* Greuter

*Hypericum kelleri* Bald.

*Hypericum trichocaulon* Boiss. & Heldr.

**Iridaceae**

*Crocus oreocreticus* B. L. Burtt

*Crocus sieberi* J. Gay

**Lamiaceae**

*Calamintha cretica* (L.) Lam.

*Micromeria hispida* Boiss. & Heldr. ex Benth.

*Micromeria sphaciotica* Boiss. & Heldr. ex Benth.

*Nepeta sphaciotica* P. H. Davis

*Origanum dictamnus* L.

*Origanum microphyllum* (Benth.) Vogel

*Phlomis lanata* Willd.

*Prunella cretensis* Gand.

*Scutellaria hirta* Sm.

*Scutellaria sieberi* Benth.

*Sideritis syriaca* L. subsp. *syriaca*

*Teucrium alpestre* Sm.

*Teucrium cuneifolium* Sm.

*Thymbra calostachya* (Rech. f.) Rech. f.

**Liliaceae**

*Fritillaria messanensis* subsp. *sphaciotica* (Gand.) Kamari & Phitos

*Gagea omalensis* J.-M. Tison

*Tulipa bakeri* A. D. Hall

*Tulipa cretica* Boiss. & Heldr.

*Tulipa doerfleri* Gand.

**Linaceae**

*Linum caespitosum* Sm.

*Linum doerfleri* Rech. f.

**Orchidaceae**

*Anacamptis papilionacea* subsp. *alibertis* (G. Kretzschmar & H. Kretzschmar) H. Kretzschmar & al.

*Cephalanthera cucullata* Boiss. & Heldr.

*Epipactis cretica* Kalop. & Robatsch

*Himantoglossum samariense* C. Alibertis & A. Alibertis

*Orchis sitiaca* (Renz) P. Delforge

*Orchis spitzelii* subsp. *nitidifolia* (W. P. Teschner) Soó

*Serapias cordigera* subsp. *cretica* B. Baumann & H. Baumann

**Plumbaginaceae**

*Acantholimon androsaceum* (Jaub. & Spach) Boiss.

*Limonium calliopsium* A. Mayer

*Limonium chersonesum* Erben & Brullo

*Limonium chrisianum* Brullo & Guarino

*Limonium cornarianum* Kypr. & R. Artelari

*Limonium creticum* R. Artelari

*Limonium elaphonisicum* A. Mayer

*Limonium grabusae* Erben & Brullo

*Limonium hierapetrae* Rech. f.

*Limonium minoicum* Erben & Brullo

*Limonium recticaule* Erben & Brullo

*Limonium sougiae* Erben & Brullo

*Limonium xerocamposicum* Erben & Brullo

**Poaceae**

*Brachypodium sylvaticum* subsp. *creticum* H. Scholz & Greuter

*Catapodium borgesii* H. Scholz

*Dactylis glomerata* L. subsp. *rigida* (Boiss. & Heldr.) Hayek

*Festuca polita* subsp. *cretica* (Markgr.-Dann.) Foggi & H. Scholz

*Sesleria doerfleri* Hayek

**Polygonaceae**

*Polygonum idaeum* Hayek

**Primulaceae**

*Cyclamen confusum* (Grey-Wilson) Culham & al.

*Cyclamen graecum* subsp. *candicum* Ietsw. ex Grey-Wilson

**Ranunculaceae**

*Adonis cretica* (Huth) Runemark

*Clematis elisabethae-carolae* Greuter

*Ranunculus cupreus* Boiss. & Heldr.

*Ranunculus radinotrichus* Greuter & Strid

*Ranunculus veronicae* N. Böhling

**Rosaceae**

*Cotoneaster creticus* J. Fryer & B. Hylmö

*Sanguisorba cretica* Hayek

**Rubiaceae**

*Asperula crassula* Greuter & Zaffran

*Asperula idaea* Halácsy

*Asperula pubescens* (Willd.) Ehrend. & Schönb.-Tem.

*Asperula rigida* Sm.

*Galium extensum* Krendl

*Galium fruticosum* Willd.

*Galium incanum* subsp. *creticum* Ehrend.

*Galium incrassatum* Halácsy

*Galium incurvum* Sm.

**Santalaceae**

*Viscum album* subsp. *creticum* N. Böhling & al.

**Scrophulariaceae**

*Verbascum arcturus* L.

*Verbascum spinosum* L.

**Tamaricaceae**

*Tamarix minoa* J. L. Villar & al.

**Ulmaceae**

*Zelkova abelicea* (Lam.) Boiss.

**Valerianaceae**

*Centranthus sieberi* Heldr.

**Veronicaceae**

*Chaenorhinum idaeum* Rech. f.

**Violaceae**

*Viola alba* subsp. *cretica* (Boiss. & Heldr.) Marcussen

*Viola fragrans* Sieber
