# Supplementary material for: Endemic plants of Crete in electronic trade and wildlife tourism: current patterns and implications for conservation
Source: J Biol Res (Thessalon). 2019 Oct 30;26:10. doi: 10.1186/s40709-019-0104-z (PMC6822446; doi:10.1186/s40709-019-0104-z)
Supplement: Supplementary file 2 — Additional file 2. Electronic addresses of the nurseries involved in the trade of Cretan endemic plants and countries where these are located. [file 40709_2019_104_MOESM2_ESM.docx]

**Additional file 2.** Electronic addresses of the nurseries involved in the trade of Cretan endemic plants and countries where these are located.

***Australia***

- [www.hillviewrareplants.com.au/nursery-catalogues/buried-treasures](http://www.hillviewrareplants.com.au/nursery-catalogues/buried-treasures)
- [www.gardenexpress.com.au/product/erysimum-bowles-mauve](http://www.gardenexpress.com.au/product/erysimum-bowles-mauve/)

***Belgium***

- [www.en.coolplants.com/catalog/index.cfm?fuseaction=shopitem&plt_id=4381](http://www.en.coolplants.com/catalog/index.cfm?fuseaction=shopitem&plt_id=4381)

***Canada***

- [www.reticulatas.com/Documents/Janis%20Ruksans%202011%20catalog.pdf](http://www.reticulatas.com/Documents/Janis%20Ruksans%202011%20catalog.pdf)
- [www.richters.com/Web_store/web_store.cgi?searchterm=origanum+dictamnus&search_catalogue_button=Go](http://www.richters.com/Web_store/web_store.cgi?searchterm=origanum+dictamnus&search_catalogue_button=Go)

***France***

- http://b-and-t-world-seeds.com/cart_print.asp?species=Draba%20cretica&sref=85735,
- [www.lepage-vivaces.com/selling.php?ONSELL=1&action=search&CONCAT_COLLECTION=10342](http://www.lepage-vivaces.com/selling.php?ONSELL=1&action=search&CONCAT_COLLECTION=10342)
- [www.pepiniere-armalette.fr/catalogues/vivaces/item/petromarula-pinnata-2.html](http://www.pepiniere-armalette.fr/catalogues/vivaces/item/petromarula-pinnata-2.html))
- [www.pepiniere-ellebore.fr/Rubriques/Catalogue/index.php?let=O&genre=ORIGANUM,edicinal%20Herbs](http://www.pepiniere-ellebore.fr/Rubriques/Catalogue/index.php?let=O&genre=ORIGANUM,edicinal%20Herbs)
- [www.senteursduquercy.com/recherche?controller=search&orderby=position&orderway=desc&search_query=phlomis&submit_search=OK](http://www.senteursduquercy.com/recherche?controller=search&orderby=position&orderway=desc&search_query=phlomis&submit_search=OK)

***Germany***

- [www.rareplants.de/shop/product.asp?strParents=0,70&CAT_ID=993&P_ID=13029](http://www.rareplants.de/shop/product.asp?strParents=0,70&CAT_ID=993&P_ID=13029)
- [www.sunshine-seeds.de/Petromarula-pinnata-55755p.html?language=en](http://www.sunshine-seeds.de/Petromarula-pinnata-55755p.html?language=en)

***Greece***

- http://www.ebay.com/itm/Petromarula-pinnata-CRETAN-ROCK-LETTUCE-Flower-Seeds-EZ-Pretty-EDIBLE-/381379917020

***Ireland***

- [www.timpanynurseries.com/Catalogue.html](http://www.timpanynurseries.com/Catalogue.html)
- [www.alpinegardensociety.ie/sources/Aberconwy%20Nursery%20-%20Spring%202014.pdf](http://www.alpinegardensociety.ie/sources/Aberconwy%20Nursery%20-%20Spring%202014.pdf)

***Italy***

- [www.vivaipriola.com/calamintha-cretica](http://www.vivaipriola.com/calamintha-cretica/)

***New Zealand***

- [www.marshwoodgardens.co.nz/search.php](http://www.marshwoodgardens.co.nz/search.php)

***Slovenia***

- [www.kpr-eshop.eu/en/bulbs/?slist_page=0&slist_letter=C](http://www.kpr-eshop.eu/en/bulbs/?slist_page=0&slist_letter=C)

***Spain***

- [www.rareplants.es/shop/page.asp?id=Aroids](http://www.rareplants.es/shop/page.asp?id=Aroids)

***The Netherlands***

- [www.green-ice-nursery.nl/pages/seedlist.html](http://www.green-ice-nursery.nl/pages/seedlist.html)

***UK***

- [www.ashwoodnurseries.com/shop/cyclamen-graecum-candicum.html](http://www.ashwoodnurseries.com/shop/cyclamen-graecum-candicum.html)
- [www.avonbulbs.co.uk/autumn-planted-bulbs/tulip-bulbs/species-and-dwarf-tulips/tulipa-bakeri-lilac-wonder](http://www.avonbulbs.co.uk/autumn-planted-bulbs/tulip-bulbs/species-and-dwarf-tulips/tulipa-bakeri-lilac-wonder)
- [www.bluebellnursery.com/catalogue/trees/Zelkova](http://www.bluebellnursery.com/catalogue/trees/Zelkova)
- [www.botanicaplantnursery.co.uk/erysimum-mutabile-4748-p.asp](http://www.botanicaplantnursery.co.uk/erysimum-mutabile-4748-p.asp)
- [www.botanyplants.co.uk/plants/](http://www.botanyplants.co.uk/plants/)
- [www.brightonplants.blogspot.gr/2009/06/phlomis-lanata.html](http://www.brightonplants.blogspot.gr/2009/06/phlomis-lanata.html)
- http://www.chilternseeds.co.uk/item.php?id=428K
- [www.crocus.co.uk/plants/_/tulipa-saxatilis-bakeri-group-lilac-wonder/classid.2000016111](http://www.crocus.co.uk/plants/_/tulipa-saxatilis-bakeri-group-lilac-wonder/classid.2000016111/)
- [www.ellisplants.co.uk/advanced_search_result.php?keywords=phlomis&osCsid=t7d4ttbnerfklkvkfoel20t995&x=7&y=10](http://www.ellisplants.co.uk/advanced_search_result.php?keywords=phlomis&osCsid=t7d4ttbnerfklkvkfoel20t995&x=7&y=10)
- [www.hillclosegardens.com/wp-content/uploads/2016/01/plantnurserystocklist.pdf](http://www.hillclosegardens.com/wp-content/uploads/2016/01/plantnurserystocklist.pdf)
- [www.jacquesamandintl.com/product/bakeri-lilac-wonder-agm](http://www.jacquesamandintl.com/product/bakeri-lilac-wonder-agm/)
- http://www.kevockgarden.co.uk/catalogue_bulbs_extra_2012.pdf
- [www.langthorns.com/product/product&product_id=9870](http://www.langthorns.com/product/product&product_id=9870)
- [www.malletcourt.co.uk/catalogue.html](http://www.malletcourt.co.uk/catalogue.html)
- [www.mountvenusnursery.com/catalog/product-html/Erysimum-mutabile-P-367.html](http://www.mountvenusnursery.com/catalog/product-html/Erysimum-mutabile-P-367.html)
- [www.plant-world-seeds.com/store/view_seed_item/3140](http://www.plant-world-seeds.com/store/view_seed_item/3140)
- [www.pottertons.co.uk/pott/find.php](http://www.pottertons.co.uk/pott/find.php)
- [www.poyntzfieldherbs.co.uk/cataloguemed1.asp?sortby=engname&colhead=cookormed&req=m&doctitle=M](http://www.poyntzfieldherbs.co.uk/cataloguemed1.asp?sortby=engname&colhead=cookormed&req=m&doctitle=M)
- [www.rareplants.co.uk/product/arum-idaeum](http://www.rareplants.co.uk/product/arum-idaeum/)
- [www.rosecottageplants.co.uk/tulipa-bakeri-lilac-wonder/p525](http://www.rosecottageplants.co.uk/tulipa-bakeri-lilac-wonder/p525)
- [www.rvroger.co.uk/index.php?linksource=stockitem&listgroupfile=bulbs&parentpagefile=autumnbulbs&season=BAUT&webfilename=muscari_spreitzenhoferi](http://www.rvroger.co.uk/index.php?linksource=stockitem&listgroupfile=bulbs&parentpagefile=autumnbulbs&season=BAUT&webfilename=muscari_spreitzenhoferi)
- [www.sarahraven.com/flowers/bulbs/tulips/tulipa_bakeri_saxatilis.htm](http://www.sarahraven.com/flowers/bulbs/tulips/tulipa_bakeri_saxatilis.htm)
- [www.talevalleynursery.co.uk/catalogueofbulbsformailorder.shtml](http://www.talevalleynursery.co.uk/catalogueofbulbsformailorder.shtml)
- [www.tortworthplants.co.uk/ourshop/prod_3707307-Tulipa-saxatilis-Bakeri-Group-Lilac-Wonder-9cm-pot.html](http://www.tortworthplants.co.uk/ourshop/prod_3707307-Tulipa-saxatilis-Bakeri-Group-Lilac-Wonder-9cm-pot.html)
- [www.tuckermarshplants.co.uk/product_page.php?id=102](http://www.tuckermarshplants.co.uk/product_page.php?id=102)

***USA***

- [www.anniesannuals.com/plants/view/?id=2007](http://www.anniesannuals.com/plants/view/?id=2007)
- [www.arrowheadalpines.com/shop/index.php?main_page=product_info&products_id=1359](http://www.arrowheadalpines.com/shop/index.php?main_page=product_info&products_id=1359)
- [www.bulbmeister.com/flowershop/catalog/fso2004-catalog.pdf](http://www.bulbmeister.com/flowershop/catalog/fso2004-catalog.pdf)
- [www.cistus.com/mail_order/information.html](http://www.cistus.com/mail_order/information.html)
- [www.companionplants.com/catalog/index.php?dir=od-oz](http://www.companionplants.com/catalog/index.php?dir=od-oz)
- [www.davidsongreenhouse.com/garden-guide.htm](http://www.davidsongreenhouse.com/garden-guide.htm)
- [www.edelweissperennials.com/Large_Image.aspx?plantName=1105](http://www.edelweissperennials.com/Large_Image.aspx?plantName=1105)
- [www.edgewoodgardens.net/cyclamen_sales.html](http://www.edgewoodgardens.net/cyclamen_sales.html)
- [www.farreachesfarm.com/SearchResults.asp?Search=phlomis&Submit=GO](http://www.farreachesfarm.com/SearchResults.asp?Search=phlomis&Submit=GO),
- [www.forestfarm.com/product.php?id=5911](http://www.forestfarm.com/product.php?id=5911)
- [www.hardyplants.com/flower/Campanula.html](http://www.hardyplants.com/flower/Campanula.html)
- [www.johnscheepers.com/tulipa-saxatilis.html](http://www.johnscheepers.com/tulipa-saxatilis.html)
- [www.morningsunherbfarm.com/product_info.php?products_id=263](http://www.morningsunherbfarm.com/product_info.php?products_id=263)
- [www.mountainvalleygrowers.com/oridictamnus.htm](http://www.mountainvalleygrowers.com/oridictamnus.htm)
- [www.mulberrycreek.com/cgi-bin/herbman/page.cgi?p=a_z;letter=O](http://www.mulberrycreek.com/cgi-bin/herbman/page.cgi?p=a_z;letter=O)
- [www.odysseybulbs.com/](http://www.odysseybulbs.com/) (United States of America)
- [www.plantlust.com/plants/42982/helichrysum-heldreichii-hythe-form](http://www.plantlust.com/plants/42982/helichrysum-heldreichii-hythe-form/)
- [www.seedhunt.com](http://www.seedhunt.com/)
- [www.urbantreefarm.com/phlomis-lanata.html](http://www.urbantreefarm.com/phlomis-lanata.html)
- [www.vanengelen.com/tulipa-saxatilis.html](http://www.vanengelen.com/tulipa-saxatilis.html)
