# Supplementary material for: Endemic plants of Crete in electronic trade and wildlife tourism: current patterns and implications for conservation
Source: J Biol Res (Thessalon). 2019 Oct 30;26:10. doi: 10.1186/s40709-019-0104-z (PMC6822446; doi:10.1186/s40709-019-0104-z)
Supplement: Supplementary file 3 — Additional file 3. Endemic taxa of Crete that are traded by nurseries via the internet or advertised by tourist agencies to attract visitors. [file 40709_2019_104_MOESM3_ESM.docx]

**Additional file 3.** Endemic taxa of Crete that are traded by nurseries via the internet or advertised by tourist agencies to attract visitors. Given are the numbers of these nurseries and agencies and also the number of countries (in parentheses) where these are located. For the taxa that have been assessed, the latest IUCN extinction risk assignment is given in brackets. Numbers as superscripts to the left of taxa correspond to the sources for current [EN: Endangered, VU: Vulnerable, NT: Near Threatened] and older [R: Rare] IUCN extinction risk assignments; these are 1 = Kozlowski et al., 2012 [55]; 2 = Bilz et al., 2011 [54]; 3 = Phitos et al., 2009 [53]; 4 = Walter and Gillett, 1998 [52]; 5 = Phitos et al., 1995 [51]. Marked with ‘*’ are bulbous/tuberous plants (geophytes), with ‘D’ are taxa included in the Annex II of Directive 92/43/EEC, with ‘B’ are taxa included in Bern Convention, with ‘C’ are taxa included in CITES, and with ‘P’ taxa included in the Greek Presidential Decree 67/1981. The symbol ‘-’ signifies absence of the taxon from the respective list of nurseries or of tourist agencies.

| **Cretan endemic plants** | **Number of nurseries (countries) involved** | **Number of tourist agencies (countries) involved** |
| --- | --- | --- |
| **Amaryllidaceae** |  |  |
| **Allium circinnatum* Sieber subsp*. circinnatum* (P) | - | 1 (1) |
| **Apiaceae** |  |  |
| *Ferulago thyrsiflora* (Sm.) W. D. J. Koch (P) | - | 1 (1) |
| **Araceae** |  |  |
| **Arum idaeum* Coustur. & Gand. | 1 (1) | 2 (1) |
| *^2^*Biarum davisii* Turrill [NT] (P) | 3 (2) | - |
| *^4^*Biarum tenuifolium* subsp*. idomenaeum* P. C. Boyce & Athanasiou [R] | 1(1) | - |
| **Asparagaceae** |  |  |
| *^3^*Bellevalia brevipedicellata* Turrill [EN] (P) | 2 (2) | - |
| **Muscari spreitzenhoferi* (Heldr. ex Osterm.) H. R. Wehrh. | 4 (2) | 4 (1) |
| **Scilla nana* subsp. *nana* (Schult. & Schult. f.) Speta (P) | - | 2 (2) |
| **Asteraceae** |  |  |
| *Centaurea idaea* Boiss. & Heldr. | - | 1 (1) |
| *^3^Helichrysum heldreichii* Boiss. [NT] (P) | 3 (1) | - |
| **Boraginaceae** |  |  |
| *^4^Anchusa cespitosa* Lam. [R] (P) | 2 (2) | 1 (1) |
| **Brassicaceae** |  |  |
| *Draba cretica* Boiss. & Heldr. | 2 (2) | - |
| *Erysimum mutabile* Boiss. & Heldr. | 6 (3) | - |
| *Erysimum raulinii* Boiss. | - | 1 (1) |
| *Ricotia cretica* Boiss. & Heldr. | - | 3 (1) |
| **Campanulaceae** |  |  |
| *^4^Campanula cretica* (A. DC.) D. Dietr. [R] (P) | 3 (3) | - |
| *Campanula tubulosa* Lam. | - | 4 (1) |
| *Petromarula pinnata* (L.) A. DC. | 5 (3) | 5 (1) |
| **Caryophyllaceae** |  |  |
| *Cerastium scaposum* Boiss. & Heldr. subsp. *scaposum* | - | 2 (1) |
| *^4^Dianthus fruticosus* subsp*. creticus* (Tausch) Runemark [R] (P) | - | 1 (1) |
| *^3^Dianthus xylorrhizus* Boiss. & Heldr. [VU] (P) | - | 1 (1) |
| **Colchicaceae** |  |  |
| **Colchicum cretense* Greuter | 1(1) | - |
| **Euphorbiaceae** |  |  |
| *^3^Euphorbia sultan-hassei* Strid & al. [NT] | - | 1(1) |
| **Fabaceae** |  |  |
| *Ebenus cretica* L. | 2 (2) | 4 (1) |
| **Fumariaceae** |  |  |
| *^4^Corydalis uniflora* (Sieber) Nyman [R] (P) | - | 2 (1) |
| **Hypericaceae** |  |  |
| *Hypericum trichocaulon* Boiss. & Heldr. (probably *H. kelleri* Bald. in UK nurseries) | 2 (2) | - |
| **Iridaceae** |  |  |
| *^4^*Crocus oreocreticus* B. L. Burtt [R] (P) | 3 (3) | - |
| **Crocus sieberi* J. Gay | - | 2(2) |
| **Lamiaceae** |  |  |
| *^4^Calamintha cretica* (L.) Lam. [R] (P) | 3 (3) | - |
| *^2^Origanum dictamnus* L. [NT] (D, B, P) | 7 (5) | 1 (1) |
| *Origanum microphyllum* (Benth.) Vogel | 3 (3) | - |
| *Phlomis lanata* Willd. (P) | 8 (4) | 1 (1) |
| *Scutellaria sieberi* Benth. | - | 4 (1) |
| *Sideritis syriaca* L. subsp*. syriaca* | 3 (3) | - |
| *^4^Teucrium alpestre* Sm. [R] | - | 1 (1) |
| **Liliaceae** |  |  |
| *Fritillaria messanensis* subsp*. sphaciotica* (Gand.) Kamari & Phitos | 1 (1) | 1 (1) |
| **Tulipa bakeri* A. D. Hall (P) | 14 (4) | 4 (2) |
| **Tulipa cretica* Boiss. & Heldr. (P) | 3 (1) | 2 (2) |
| *^4^*Tulipa doerfleri* Gand. [VU] (P) | - | 7 (2) |
| **Orchidaceae** |  |  |
| *Anacamptis papilionacea* subsp*. alibertis* (G. Kretzschmar & H. Kretzschmar) H. Kretzschmar & al. (P) | - | 1 (1) |
| *^2^Cephalanthera cucullata* Boiss. & Heldr. [EN] (D, B, P) | - | 2 (2) |
| *Himantoglossum samariense* C. Alibertis & A. Alibertis (P) | - | 1 (1) |
| *^2^Orchis sitiaca* (Renz) P. Delforge [EN] (P) | - | 1 (1) |
| **Plumbaginaceae** |  |  |
| *^5^Acantholimon androsaceum* (Jaub. & Spach) Boiss. [VU] | 3 (3) | - |
| **Primulaceae** |  |  |
| *^*^Cyclamen confusum* (Grey-Wilson) Culham & al. (C) | 4 (2) | - |
| *^*^Cyclamen graecum* subsp*. candicum* Ietsw. ex Grey-Wilson (C) | 3 (1) | - |
| **Ranunculaceae** |  |  |
| *Adonis cretica* (Huth) Runemark in Strid & Tan | - | 1 (1) |
| *^4^Ranunculus cupreus* Boiss. & Heldr. [VU] (P) | - | 1 (1) |
| **Rosaceae** |  |  |
| *^4^Sanguisorba cretica* Hayek [R] (P) | - | 1 (1) |
| **Rubiaceae** |  |  |
| *Asperula pubescens* (Willd.) Ehrend. & Schönb.-Tem. | - | 1(1) |
| **Scrophulariaceae** |  |  |
| **Verbascum arcturus* L. | 5 (5) | 6 (2) |
| **Ulmaceae** |  |  |
| *^1^Zelkova abelicea* (Lam.) Boiss. [EN] (D, B, P) | 2 (1) | 3 (1) |
| **Violaceae** |  |  |
| *Viola alba* subsp. *cretica* (Boiss. & Heldr.) Marcussen (P) | - | 1 (1) |
