# Supplementary material for: Endemic plants of Crete in electronic trade and wildlife tourism: current patterns and implications for conservation
Source: J Biol Res (Thessalon). 2019 Oct 30;26:10. doi: 10.1186/s40709-019-0104-z (PMC6822446; doi:10.1186/s40709-019-0104-z)
Supplement: Supplementary file 4 — Additional file 4. Prices in Euros (US Dollars, in parenthesis) of the Cretan endemic taxa that are traded by nurseries via the Internet as living plants, bulbs and seeds. The exchange rate for Euros and Dollars are as of July 17, 2017 (1 € = 1.15 $). [file 40709_2019_104_MOESM4_ESM.docx]

| **Additional file 4.** Prices in Euros (US Dollars, in parenthesis) of the Cretan endemic taxa that are traded by nurseries via the Internet as living plants, bulbs and seeds. The exchange rate for Euros and Dollars are as of July 17, 2017 (1 € = 1.15 **$**). | | | | | |
| --- | --- | --- | --- | --- | --- |
| **Taxa** | **Price in EUR, € (USD, $)** | | | | |
|  | **Living**  **plant** | **Individual**  **bulb** | **Individual seed** | **Seed**  **pack** | **Seeds per gram** |
| 1. *Acantholimon androsaceum* (Jaub. & Spach) Boiss. | 3.50 (4.02), 3.43 (3.92), 3.43 (3.92) |  |  |  |  |
| 2. *Anchusa cespitosa* Lam. | 5.71 (6.50), 16.60 (19.00) |  |  |  |  |
| 3. *Arum idaeum* Coustur. & Gand. | 12.00 (13.72) |  |  |  |  |
| 4. *Bellevalia brevipedicellata* Turrill |  | 6.07 (6.95), 10.28 (11.76) |  |  |  |
| 5. *Biarum davisii* Turrill | 6.99 (8.00), 8.58 (9.80) | 11.43 (13.1) |  |  |  |
| 6. *Biarum tenuifolium* subsp*. idomenaeum* P. C. Boyce & Athanasiou |  | 15.99 (18.29) |  |  |  |
| 7. *Calamintha cretica* (L.) Lam | 3.00 (3.45) |  | 0.02 (0.023) | 4.99 (5.74) |  |
| 8. *Campanula cretica* (A. DC.) D. Dietr. | 2.58 (2.95) |  |  | 2.84 (3.27),  4.00 (4.60) |  |
| 9. *Colchicum cretense* Greuter |  | 18.00 (20.7) |  |  |  |
| 10. *Crocus oreocreticus* B. L. Burtt |  | 3.49 (4.00), 1.26 (1.44) | 0.52 (0.60) |  |  |
| 11. *Cyclamen confusum* (Grey-Wilson) Culham & al. | 6.76 (7.75), 4.61 (5.29),  10.47 (12.00), 6.98 (8.00) |  | 0.24 (0.28) |  |  |
| 12. *Cyclamen graecum* subsp*. candicum* Ietsw.ex Grey-Wilson | 6.28 (7.18), 10.48 (12.00) |  | 0.30 (0.35) |  |  |
| 13. *Draba cretica* Boiss. & Heldr. |  |  | 0.22 (0.25) |  | 45.00 (51.75) |
| 14. *Ebenus cretica*  L. |  |  | 0.35 (0.40) | 14.9 (17.14) |  |
| 15. *Erysimum mutabile* Boiss. & Heldr. | 7.43 (8.50), 6.50 (7.47), 5.94 (6.80),  9.13 (10.44), 3.43 (3.92), 4.57 (5.22) |  |  |  |  |
| 16. *Fritillaria messanensis* subsp*. sphaciotica* (Gand.) Kamari & Phitos |  |  | 0.52 (0.60) |  |  |
| 17. *Helichrysum heldreichii* Boiss. | 7.82 (8.95),8.74 (10.00), 9.61 (11.00) |  |  |  |  |
| 18. *Hypericum trichocaulon* Boiss. & Heldr. (probably *H. kelleri* Bald. in UK nurseries) | 4.57 (5.22) |  |  | 4.00 (4.60) |  |
| 19. *Muscari spreitzenhoferi* (Heldr. ex Osterm.) H. R. Wehrh. |  | 10.22 (11.69), 6.28 (7.18), 10.28 (11.76), 6.00 (6.90) |  |  |  |
| 20. *Origanum dictamnus* L. | 2.84 (3.25), 4.18 (4.81), 5.02 (5.75),  4.8 (5.50), 8.00 (9.20), 7.31 (8.36) |  |  |  |  |
| 21. *Origanum microphyllum* (Benth.) Vogel | 3.48 (3.99), 4.00 (4.60) |  |  |  |  |
| 22. *Petromarula pinnata* (L.) A. DC. | 6.94 (7.95), 10.00 (11.5) |  | 0.13 (0.15), 0.07 (0.08) | 2.50 (2.88) |  |
| 23. *Phlomis lanata* Willd. | 6.79 (7.77), 6.90 (7.93), 5.20 (5.95),  5.50 (6.33), 9.13 (10.45), 6.11 (7.00),  7.86 (9.00), 10.48 (12.00) |  |  |  |  |
| 24. *Sideritis syriaca* L. subsp*. syriaca* |  |  |  | 3.45 (4.00),  7.99 (9.20) | 0.07 (0.08) |
| 25. *Tulipa bakeri* A. D. Hall |  | 0.35 (0.40), 1.80 (2.05), 0.31 (0.35), 0.16 (0.18), 0.32 (0.37), 0.38 (0.43), 0.23 (0.26),0.46 (0.52), 0.29 (0.33), 0.34 (0.39), 0.20 (0.23) |  | 2.28 (2.60),  5.65 (6.47),  4.99 (5.74) |  |
| 26. *Tulipa cretica* Boiss. & Heldr. |  | 4.18 (4.81), 4.57 (5.25), 3.43 (3.95) |  |  |  |
| 27. *Verbascum arcturus* L. | 6.93 (7.95) |  | 0.03 (0.04), 0.12 (0.14) | 3.19 (3.66),  7.99 (9.20) |  |
| 28. *Zelkova abelicea* (Lam.) Boiss. | 33.61 (38.54), 39.88 (45.73) |  |  |  |  |
