# Supplementary material for: Endemic plants of Crete in electronic trade and wildlife tourism: current patterns and implications for conservation
Source: J Biol Res (Thessalon). 2019 Oct 30;26:10. doi: 10.1186/s40709-019-0104-z (PMC6822446; doi:10.1186/s40709-019-0104-z)
Supplement: Supplementary file 5 — Additional file 5. Electronic addresses of tourist agencies using Cretan endemic plants to attract tourists, and countries where these are located. [file 40709_2019_104_MOESM5_ESM.docx]

**Additional file 5.** Electronic addresses of tourist agencies using Cretan endemic plants to attract tourists, and countries where these are located.

***Greece***

| - <https://www.argonautravel.com> |
| --- |
| - http://www.flowersofcrete.info/trips2.html |
| - [http://greentour.gr/wildlife-nature/wild-flowers](http://greentour.gr/wildlife-nature/wild-flowers/) |
| - <http://www.travelcrete.gr/en/tour/crete-by-invitation> |
| ***UK***   - <http://www.naturetrek.co.uk/tour.aspx?id=182> |
| - <http://www.honeyguide.co.uk/wildlife-holidays/crete.html> |
| - [http://greenwings.co/our-holidays/botanical-holidays/flowers-crete](http://greenwings.co/our-holidays/botanical-holidays/flowers-crete/) |
| - [http://www.greentours.co.uk/Europe/CRETE](http://www.greentours.co.uk/Europe/CRETE/) |
| - [http://wildlife-travel.co.uk](../../../Despoina%20Vokou/AppData/Local/Temp/Paper%20final/Ειδη%20Κρητης%20-%20Κυπρου%20από%20εκδρομε.xls) |
